# Total thyroidectomy vs completion thyroidectomy for thyroid nodules with indeterminate cytology/follicular proliferation: a single-centre experience

**DOI:** 10.1186/s12893-019-0552-2

**Published:** 2019-07-10

**Authors:** Giuseppe Sena, Gaetano Gallo, Nadia Innaro, Noemi Laquatra, Martina Tolone, Rosario Sacco, Giuseppe Sammarco

**Affiliations:** Department of Health Sciences, U.O. of Digestive Surgery, University of Catanzaro, Viale Europa, 88100 Catanzaro, Italy

**Keywords:** Total thyroidectomy, Completion thyroidectomy, Complication rates, Differentiated thyroid cancer

## Abstract

**Background:**

Despite total thyroidectomy (TT) is the most practiced procedure for a preoperatively diagnosed neoplastic lesion, according to the ATA guidelines, many surgeons perform completion thyroidectomy (CT) after hemithyroidectomy for patients with preoperative follicular proliferation/indeterminate cytology who are diagnosed with malignancy. CT has a higher complication rate than the primary procedure. The primary endpoint of our study is to compare the morbidity rate after CT with that after primary TT in patients with follicular proliferation/indeterminate cytology.

**Methods:**

We retrospectively reviewed 237 patients who underwent thyroid surgery from 2009 to 2018 at our institution. We recruited only patients with follicular proliferation/indeterminate cytology and excluded those undergoing lymphadenectomies and thyroidectomies for benign pathology and staged thyroidectomies after intraoperative documentation of a RLN lesion. One hundred eighty-six of these patients underwent TT, and fifty-one underwent CT for the detection of differentiated thyroid cancer at the histological exam.

**Results:**

No differences were found in the total complication rates between the two groups (OR 0,76, 95% CI 0.35–1.65, *P* = 0.49). We did not find any significant differences in the subgroup analysis. In particular, no significant differences were identified for transient hypocalcaemia (OR 1.17, 95% CI 0.44–3.11; *P* = 0,74), permanent hypocalcaemia (OR 1.04, 95% CI 0.21–5.18; P = 0,95), transient unilateral recurrent laryngeal nerve palsy (OR 0.78, 95% CI 0.21–2.81; P = 0,16), permanent unilateral recurrent laryngeal nerve palsy (OR 1.48, 95% CI 0.28–7.85; P = 0,61), and haematoma (OR 1,84, 95% CI 0,16-20,71; P = 0,61).

**Conclusions:**

CT following hemithyroidectomy can be performed with acceptable morbidity in patients with thyroid nodules with preoperative indeterminate cytology/follicular proliferation.

## Background

Differentiated thyroid carcinoma (DTC) is one of the most frequent malignancies but fortunately has an optimal prognosis [1–3]. Surgical therapy is mandatory for resolution of this condition. Unfortunately, surgical treatment of thyroid diseases is still controversial. Total thyroidectomy (TT) has been considered the best treatment for DTC with behaviours of multicentricity and bilaterality for a long time because it eliminates the potential residual tumour risk and allows better ablation of possible residual tissue with radioactive iodine [4–8]. Despite TT is the most practiced procedure for a preoperatively diagnosed neoplastic lesion, according to the ATA guidelines, many surgeons perform completion thyroidectomy (CT) after hemithyroidectomy for patients with preoperative follicular proliferation/indeterminate cytology who are diagnosed with malignancy [9–11]. CT has a higher complication rate than the primary procedure due to adhesions and the distorted anatomy following initial neck exploration [12, 13]. Main complications are temporary recurrent laryngeal nerve (RLN) palsy, permanent RLN palsy, temporary hypocalcaemia, permanent hypocalcaemia, haematoma, and wound infection. However, recent improvements in surgical techniques have reduced the complication rate. Therefore, the primary endpoint of our study is to compare the morbidity rate after CT with the morbidity rate after TT in patients with follicular proliferation/indeterminate cytology and to compare these results with published data.

## Methods

In this retrospective study, we reviewed 237 patients who underwent TT or completion thyroidectomy at our institution from 2009 to 2018. We recruited only patients with follicular proliferation/indeterminate cytology and excluded those undergoing lymphadenectomies, thyroidectomies for benign diseases and staged thyroidectomies after intraoperative documentation of a RLN lesion. One hundred eighty-six of these patients underwent TT and fifty-one underwent CT for detection of DTC at the histological exam. We carried out CT three to twelve weeks after hemithyroidectomy. In all patients, we performed an US scan of the neck for assessment of the thyroid gland and locoregional nodes, measured the serum triiodothyronine (T3), thyroxin (T4), and thyroid stimulating hormone (TSH) concentrations and the blood calcium level, and performed fine needle aspiration (FNA) cytology and direct fibrolaryngoscopy. Computer tomography scanning was used selectively in cases with suspected infiltration of nearby structures. We assessed serum calcium levels routinely on postoperative day one, and we repeated this measurement until discharge on the third postoperative day. For all patients, we collected age, sex, operative procedures, preoperative cytology, histological cancer type and all postoperative complications. All interventions were carried out by the same first operator (NI). We performed all the procedures using previously NIM® 2.4 system and recently NIM® 3.0 system (Medtronic, Jacksonville, USA) for intraoperative (RLN) monitoring (IONM). We used standardized technique of IONM RLNs, whit vagal assessment at the beginning and at the end of surgery. Loss of signal (LOS) was defined as absence of signal or electromyography signal amplitude below 100 μV, lack of palpable laryngeal twitch, or visible laryngeal movement, following stimulation of the ipsilateral vagus nerve [14]. We examined transient and permanent hypocalcaemia, transient and permanent recurrent laryngeal nerve (RLN) palsy, haemorrhage and minor wound infection. Permanent and transient RLN palsy was confirmed by fibrolaryngoscopy postoperatively.

### Surgical technique

TT and CT were performed using standard surgical techniques. After making a Kocher collar incision, the skin flaps were prepared, and the cervical muscular fascia was divided longitudinally along the midline of the neck. Subsequently, the upper pole was exposed and retracted, and the superior thyroid artery and vein were divided. Then, the lobe was retracted out of the wound. In this step, both the superior and inferior parathyroid glands were mobilized and retracted laterally with their vascular pedicles. As the dissection proceeded medially, the recurrent laryngeal nerve was encountered and carefully identified. Then, the terminal division branches of the inferior thyroid artery were ligated and divided. The dissection proceeded to the region of the ligament of Berry, which was divided using a Harmonic scalpel. After full mobilization of the dominant lobe, the contralateral lobe was removed, and the steps were repeated with eventual removal of the entire thyroid gland.

### Statistical analysis

All patient data were collected with a dedicated electronic Microsoft Office Excel Database (Microsoft Corp, Redmond, WA, USA). Differences between groups were analysed with the Chi-square test. Odds ratios (ORs) were reported with their 95% confidence intervals (CIs). *P* values less than 0.05 were considered significant. The statistical analysis was performed using the SPSS 10.0 software (Statistical Software, Chicago, IL, USA).

## Results

Two hundred thirty-seven patients underwent thyroid surgery from 2009 to 2018 at our institution. One hundred eighty-six patients with indeterminate results/follicular proliferation at preoperative cytology underwent TT (Group 1). This procedure was performed for high-risk patients, including those with a family history of cancer, previous neck irradiation, high-volume nodules, evidence of extracapsular invasion, and bilateral nodules. Fifty-one patients received CT after detection of DTC at the definitive histological exam (Group 2). These patients had previously underwent a hemithyroidectomy for the presence of single nodules with a low risk of cancer according to the ATA guidelines. In TT group 26 nodules were papillar carcinomas and 20 nodules were follicular carcinomas with a mean size of 18,6 mm (6–47 mm). In CT group 30 nodules were papillar carcinomas and 21 nodules were follicular carcinomas with a mean size of 15,2 mm (5–42 mm). The clinical and pathological characteristics of the patients are shown in Table 1. Intraoperative LOS was noted in 24 (12,9%) and 7 (13,7%) cases in TT and CT groups, respectively. Unilateral temporary RLN palsy rates were 7,5% (14/186) and 5,8 (3/51) in the TT and CT groups, respectively. The unilateral permanent RLN palsy occurred in 2,6% (5/186) and 3,9% (2/51) of the patients in the TT and CT groups, respectively. Temporary hypocalcaemia was noted in 19 (10,2%) and in 6 (11,7%) cases in TT and CT groups respectively. The prevalence of permanent hypocalcaemia was 3,7% (7/181) in the TT group versus 3.9% (2/51) in the CT group. The haematoma rates were 1,0% in the TT group versus 1,9% in the CT group.

**Table 1:** Clinical and Pathologic Characteristics of 237 patients

	Total Thyroidectomy	Completion Thyroidectomy	P
Patients (%)	186 (78)	51 (21)	0,0001
Mean Age	51,2	53,9	NV
Age > 50(%)	89 (37,5)	35 (68,6)	0,1
Age < 50(%)	97 (40,9)	16 (31,3)	1,6
Female (%)	158 (66)	41 (80)	0,05
Male (%)	79 (32)	10 (19)	0,05

No difference was found in the total complication rates between the two groups (OR 0,7; 95% CI 0.3–1.6, *P* = 0.4). We did not find significant differences in the subgroup analysis. The postoperative complications are shown in Table 2.

**Table 2:** Post-operative complications

	Total Thyroidectomy	Completion Thyroidectomy	Odds Ratio
Temporary Hypocalcemia (%)	19 (10,2)	6 (11.7)	1,1 95%CI (0,4-3,1) p = 0,7
Permanent Hypocalcemia (%)	7 (3,7)	2 (3,9)	1,0 95%CI (0,2-5,1) p = 0,9
Temporary RLN Palsy (Unilateral) (%)	14 (7,5)	3 (5,8)	0,7 95%CI (0,2-2,8) p = 0,1
Permanent RLN Palsy (Unilateral)(%)	5(2.6).	2 (3,9)	1,4 95%CI (0,2-7,8) p = 0,6
Hematoma (%)	2 (1,0)	1 (1,9)	1,8 95%CI (0,1-20,7)p = 0,6
Intraoperative Los (%)	24 (12,9)	7 (13,7)	0,9 95%Cl (0,3-2,3) p = 0,8

## Discussion

According to the ATA guidelines, patients with an indeterminate result or follicular proliferation at FNA cytology with low-risk and monolateral nodules often undergo unilateral hemithyroidectomy. A CT is performed when DTC is found at the final histological examination, because performing only a hemithyroidectomy is considered oncologically inadequate, and a consensus exists concerning complete removal of the lobe with malignant nodules. Many surgeons prefer to perform a thyroidectomy to increase the effectiveness of adjuvant treatments and to allow adequate follow-up [15]. CT improve oncological radicality. In fact, the rate of neoplastic disease is greater than 40% in the ipsilateral residual lobe and 30% in the contralateral lobe after completion thyroidectomy [16–18]. Because the residual disease is the most important prognostic factor, a radical resection reduces loco-regional recurrence and distance diffusion [19, 20], as well as low-risk carcinoma [21, 22]. After execution of a CT, the thyroglobulin dosage is an important prognostic indicator. Moreover, the removal of all thyroid tissue increases the effectiveness of radioiodine therapy for the elimination of any residual neoplastic foci. The same surgeon advocates the possibility of ablation of the remaining thyroid with I131 radiometabolic therapy. However, this method uses different dosage ranges and does not allow removal of remaining large glands. In addition, a high dosages of radioactive iodine favor the onset of pulmonary fibrosis, temporary bone marrow suppression, and leukaemia [23]. Therefore, this method does not result in the desired benefits. CT is traditionally characterized by a high complication rate, and complications are the reason for fear and hesitation on the part of surgeons. Complications occur due to the presence of adhesions, which make dissection difficult [12, 13]. However, the literature shows a low complication rate due to improvement surgical techniques and high-volume surgeons. In a 2015 meta-analysis that included 7 studies and 1,208 patients and compared the complication rates between CT and primary TT for differentiated thyroid cancer, Li et al. did not find statistically significant differences in the presence of temporary recurrent laryngeal nerve (RLN) palsy, permanent RLN palsy, temporary hypocalcaemia, permanent hypocalcaemia, haematoma, and wound infection. Indeed, in this work, the temporary RLN palsy occurred in 10.1% of patients in the CT group versus 8,1% in TT groups, respectively. The permanent RLN palsy was noted in 1.8% of patients in the CT (12/682) groups and 1.1% in TT (7/655) groups, respectively. The Temporary hypocalcaemia rates were 14.9% in CT group versus 15.4% in the TT group, and the rates of permanent hypocalcaemia were 2.8% in the CT group versus 3.3% in the TT group [24]. Based on our experience with 237 patients over an approximately 10-year period, we compared TT with CT only in patients with preoperative cytology showing indeterminate results/follicular proliferation of the thyroid nodule. We report a 5,8% rate of transient unilateral RLN palsy, 3,9% of permanent RLN unilateral palsy, 11,7% of transient hypocalcaemia and 3,9% of permanent hypocalcaemia in the CT group and 7,5% rate of transient unilateral RLN palsy, 2,6% of permanent RLN unilateral palsy, 10,21% of transient hypocalcaemia and 3,7% of permanent hypocalcaemia in the TT group. These results were consistent with the experiences of other centres [25–32]. Moreover, we were not able to compare our group with those underwent TT with ipsilateral or bilateral central neck dissection because our study concerned patients with follicular proliferation/indeterminate cytology excluding those who underwent lymphadenectomies and thyroidectomies for benign diseases [33]. According ATA guidelines we performed therapeutic central-compartment (level VI) neck dissection for patients with clinically involved central nodes and prophylactic central-compartment neck dissection in patients with thyroid carcinoma with clinically uninvolved central neck lymph nodes (cN0) who have advanced primary tumors (T3 or T4) or clinically involved lateral neck nodes [9]. The low rate of hypoparathyroidism in the CT group could be due to functional recovery of the parathyroid glands after the injury caused at the first operation. Indeed, several studies have shown that devascularized glands require approximately 4 weeks to return to full function [34]. Furthermore, because dissecting the scar tissue at the excised lobe site is not necessary, there is an absence of scar tissue in the remnant lobe region; therefore, the complication rate is low in CT thyroidectomies after hemithyroidectomies. Because use of a harmonic scalpel and nerve monitoring may give a slight advantage in overall outcomes [35–37], we use these devices for all patients. In a recent meta-analysis that compared neuromonitoring with recurrent laryngeal nerve visualization, no differences were identified in the transient or permanent RLN palsy rates [38]. However, data from the literature show that nerve monitoring during completion thyroidectomy may decrease the RLN palsy risk [39]. Our study is the first in the literature to compare the rate of complications in patients undergoing total thyroidectomy and completion thyroidectomy for indeterminate cytology/follicular proliferation of nodules. The retrospective nature of the study and the presence of a non-homogeneous group of patients represent the main limitations.

## Conclusions

Completion thyroidectomy following hemithyroidectomy can be performed with acceptable morbidity in patients with preoperative indeterminate cytology/follicular proliferation of thyroid nodules.

## Data Availability

The datasets used and/or analyzed during the current study available from the corresponding author on reasonable request**.** Data obtained for our study was publically availible under ‘methods’.

## Abbreviations

ATA: American Thyroid Association
CT: Completion Thyroidectomy
DTC: Differentiated Thyroid Cancer
EMG: Electromyography
IONM: Intraoperative neuro monitoring
LOS: Loss of Signal
RLN: Recurrent Laryngeal Nerve
TT: Total Thyroidectomy
